# Association of Salivary Amylase (*AMY1*) Gene Copy Number with Obesity in Alabama Elementary School Children

**DOI:** 10.3390/nu11061379

**Published:** 2019-06-19

**Authors:** Chandra M. K. Venkatapoorna, Priscilla Ayine, Emily P. Parra, Taylor Koenigs, Megan Phillips, Jeganathan R. Babu, Maninder Sandey, Thangiah Geetha

**Affiliations:** 1 Department of Nutrition, Dietetics, and Hospitality Management, Auburn University, Auburn, AL 36849, USA; iamchandru@gmail.com (C.M.K.V.); pza0022@tigermail.auburn.edu (P.A.); epp0008@tigermail.auburn.edu (E.P.P.); tnk0005@tigermail.auburn.edu (T.K.); phillml@tigermail.auburn.edu (M.P.); jeganrb@auburn.edu (J.R.B.); 2 Boshell Metabolic Diseases and Diabetes Program, Auburn University, Auburn, AL 36849, USA; 3 Department of Pathobiology, College of Veterinary Medicine, Auburn University, Auburn, AL 36849, USA; mzs0011@auburn.edu

**Keywords:** salivary amylase, *AMY1*, childhood obesity, copy number variant

## Abstract

Salivary amylase (AMY1) is the most abundant enzyme in human saliva, responsible for the hydrolysis of α-1,4 glycosidic linkages that aids in the digestion of starch. Recently studies have shown that the copy number of AMY1 is associated with obesity; however, the data varies with location. One-third of children are overweight/obese in Alabama. In this study, we aim to determine the relationship between the copy number of *AMY1* gene and obesity measurements in children from Alabama. One hundred twenty-seven children aged between 6 to 10 years participated in this study. Anthropometric measurements were measured using WHO recommendations. Genomic DNA was extracted from saliva, and the copy number of the *AMY1* gene was estimated by digital PCR. The association between *AMY1* copy number and obesity measurements was analyzed by linear regression. The mean *AMY1* copy number significantly decreased in overweight/obese (6.21 ± 1.48) compared to normal weight (7.97 ± 2.35) children. *AMY1* copy number inversely associated with the obesity measurements. African Americans had a stronger association between low *AMY1* copy number and obesity compared to white/European Americans. Our findings suggest that overweight/obese children have a low *AMY1* copy number and the effect is more prominent in African Americans.

## 1. Introduction

Obesity is a major health problem throughout the world [1]. Around 12.7 million children and adolescents are obese in United States [2]. Alabama is 6th highest ranked with obesity in United States, and 35% of children are overweight and obese [3]. The major factors contributing to the increase in prevalence of childhood obesity might be environmental conditions [4], sedentary activities [5], socioeconomic status [6], and food availability [7]; in addition to these, genetic factors could also be involved [8]. However, the genetic factors contributing to childhood obesity in elementary school children from Alabama has been poorly studied.

Copy number variations (CNV) contribute novel insights to the genetic heritability of human diseases such as autisim [9], type 2 diabetes [10], and obesity [11]. A segment of DNA expressed in a different copy number among individuals compared to a reference genome is defined as CNV [12]. In early-onset obesity, the first study reported the deletion within chromosome 16p11.2 [13], which has been particularly well studied since; deletions of this type are associated with obesity and duplications are associated with an underweight phenotype [14]. Previous studies have identified variation in the copy number of the candidate regions near the neuronal growth regulator 1 (NEGR1) locus [15], chromosome 10q11.22 [16], 11q11 [17], and 10q26.3 [18] with obesity. The salivary and pancreatic amylases (*AMY1* and *AMY2*) are secreted enzymes responsible for the hydrolysis of a-1,4 glyosidic linkages that aids in digestion of dietary starch [19]. The salivary amylase (*AMY1*) is the most abundant enzyme in human saliva, accounting for 40% to 50% of total salivary protein [20,21]. The CNV of *AMY1* ranges from two to 20 [14,22]. The copy number of *AMY1* is dependent upon the dietary habits. Specifically, individuals consuming higher levels of starch have a greater amount of *AMY1* compared to those consuming less starch [19,22]. The levels of salivary amylase protein and serum amylase is found to be correlated with the copy number [19,23]. Increased secretion of salivary amylase protein and copy number helps in the starch digestion. This suggests a genetic link between carbohydrate metabolism and obesity. The variation in the copy number is not only dependent upon the diet but also environmental factors, including stress levels and circadian rhythms [24,25,26]. In European and Asian adults, it has been shown that *AMY1* copy number is associated with obesity [23]; higher BMI is associated with lower gene copy number. Another study in Finland, Viljakainen et al. found no difference in *AMY1* copy numbers between healthy subjects and subjects with history of childhood-onset obesity, but obese men had higher copy number compared to females [27]. In children from an Italian school, BMI was negatively associated with *AMY1* copy number only in boys [28]. However, in Mexican children, all normal weight children had a *AMY1* copy number greater than 10 [29]. The results vary with location. The objective of this study was to evaluate the association between *AMY1* copy number and obesity measurements as well as racial disparity between white/European Americans and African Americans in elementary school children from Alabama. On the basis of the other studies, we hypothesized that the *AMY1* copy number would be lower in overweight/obese children compared to normal weight children and that there may be differences depending upon the race and ethnicity.

## 2. Materials and Methods 

### 2.1. Participants 

Around 127 children aged 6–10 (6.93 ± 1.79) years were recruited from Lee County and Macon County, Alabama by posting flyers. Children with major health disorders such as diabetes or cardiovascular disease based on an initial phone survey with the parents were excluded. The parents brought their child to Auburn University to participate in this study. Written consent was obtained from the parents and participants. The study was approved by the Auburn University Institutional Review Board.

### 2.2. Anthropometric Measurements

All the anthropometric measurements were carried out using WHO recommendations. The body weight was measured without shoes and light clothing using a Tanita digital scale to the nearest 4 ounces. The height was measured on a calibrated scale attached to a stadiometer to the nearest 0.1 cm [30]. The Body Mass Index (BMI) was calculated to determine the body fat and approximate the weight and height of the participants. As growth occurs until the age of 20, and not all the growth is related to body fat, BMI *z*-scores were calculated utilizing a SPSS macro based on WHO growth reference 2007 data adjusted for age and sex [31]. The Centers for Diseases Control and Prevention (CDC) standard for classification in children are: underweight (<5th percentile), normal weight (≥5th to ≤ 85th percentile), overweight (>85th to ≤ 95th percentile), and obese (>95th percentile) [32]. The recruited participants were classified as normal weight and overweight/obese based upon their percentile range. The waist circumference was determined to the nearest 0.1 cm using flexible non-elastic tape at the midpoint between the lowest ribs and the iliac crest. The *z*-scores for waist circumference (WC) and waist:height ratio (WHtR) were calculated using the R macro package developed by Sharma et al., based onLMS [Lambda (L) for the skew, Mu (M) for the median, and Sigma (S) for the generalized coefficient of variation] tables from NHANES III [33]. 

### 2.3. AMY1 Gene Copy Number

Saliva was collected from children using DNA GenoTek Saliva Collection Kit (Ontario, Canada). Genomic DNA from the saliva was extracted using the PrepIT.L2P method (DNA GenoTek, Ontario, Canada), according to the manufacturers protocol. The copy number of *AMY1* gene was estimated by digital PCR (QuantStudio™ 3D Digital PCR) containing two TaqMan assays, one for *AMY1* (Hs07226361_cn, FAM-labeled) and second, specific for the reference gene (RNase P, VIC labeled) (Life Technologies, Carlsbad, CA, USA). In brief, 14.5 μL of TaqMan PCR reaction mixture was prepared by adding 7.25 μL of QuantStudio™ 3D Digital PCR Master Mix, 0.725 μL of 20× *AMY1*, 0.725 μL of 20× RNase P, and 6 μL of diluted DNA (10 ng/μL). This reaction mixture was loaded into the QuantStudio™ 3D Digital PCR Chip, which has 20000 mini-chambers. PCR was performed using the ProFlex™ 2× Flat PCR System with the following cycling conditions. Initial denaturation at 96 °C for 10 min, 39 cycles of 60 °C for 2 min and 98 °C for 30 sec, followed by one cycle of 60 °C incubation for 2 min, and then 4 °C hold. The chip was scanned in QuantStudio™ 3D Digital PCR instrument, and subsequent analysis was performed using the QuantStudio 3D Analysis Suite Software. Hap Map sample NA18956 (Coriell Institute, Camden, NJ, USA) was used as a calibrator sample, as this sample was consistently reported to have six copies of *AMY1* by several independent methods [14,19,34]. 

### 2.4. Statistical Analysis

Data are expressed as mean ± standard deviation. Statistical analyses were performed using SPSS (version 24, IBM, Armonk, NY, USA). Independent sample *t*-test was used to assess the difference between the mean values of two groups. Non-parametric median test was used to analyze the difference between the median values of two groups. Linear regression analysis was used to investigate the association of *AMY1* copy number with BMI *z*-score, waist circumference *z*-score (WC *z*-score), and waist circumference adjusted height *z*-score (WHtR *z*-score). When considering interaction, a *p* value <0.05 was considered statistically significant. The standardized β-coefficient value was used to quantify the association. 

## 3. Results

This study cohort consisted of 127 participants (76 normal weight (NW) and 51 overweight/obese (OW/OB) children) aged between 6 to 10 years. Table 1 shows the general characteristics in the study population. The mean age of children was not statistically different between the groups. However, as expected, all anthropometric characteristics were significantly greater in OW/OB children when compared to NW children. The BMI (Kg/m^2^) of OW/OB (21.16 ± 3.15) children was significantly (*p* < 0.00001) greater compared to NW (16.03 ± 1.56) subjects. Likewise, the waist circumference of OW/OB subjects (71.06 ± 8.67) was significantly greater (*p* < 0.0001) compared to NW (60.28 ± 5.26) subjects. 

Table 2 shows the descriptive analysis of *AMY1* copy number in the study population. The copy number of *AMY1* ranged from 2.03 to 16.25 with a median of 6.93. In NW subjects, the *AMY1* copy numbers were in the range of 2.03 to 16.25 with a median of 7.835. In the OW/OB subjects, the *AMY1* copy numbers ranged from 2.95 to 10.46 with a median of 5.89. A race specific descriptive analysis of *AMY1* copy number is shown in Table 3. The *AMY1* copy number in white/European Americans (EA) ranged from 2.03 to 15.16, and in African American (AA) children it was from 2.95 to 16.25. 

**Figure 1:**
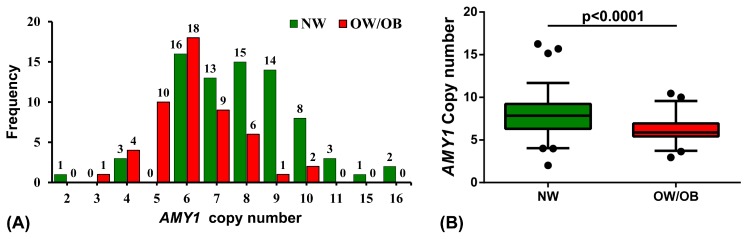
The *AMY1* copy number in the study population. (**A**) Distribution of *AMY1* copy number in the study population. The copy number of *AMY1* was rounded to nearest integer. (**B**) Mean *AMY1* copy number in normal weight (NW) and overweight/obese (OW/OB) participants (*p* < 0.0001).

**Figure 2:**
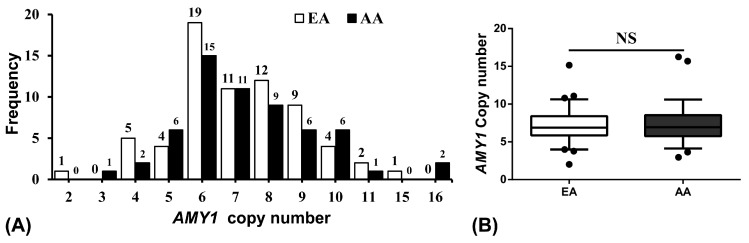
The *AMY1* copy number in White/European American (EA) and African American (AA) children. (**A**) Distribution of *AMY1* copy number in European American and African American children. The copy number of *AMY1* was rounded to nearest integer. (**B**) Mean *AMY1* copy number in European American and African American. NS—No significance.

Figure 1A shows the distribution of *AMY1* copy number in NW and OW/OB children. The median copy number of *AMY1* in OW/OB (5.89) is significantly lower (*p* < 0.0001) compared to NW (7.83) children (Figure 1B). Figure 2A shows the race specific distribution of *AMY1* copy number in the study population. The *AMY1* copy number in EA (6.89) and AA (6.94) are not statistically significant as shown in Figure 2B.

**Figure 3:**
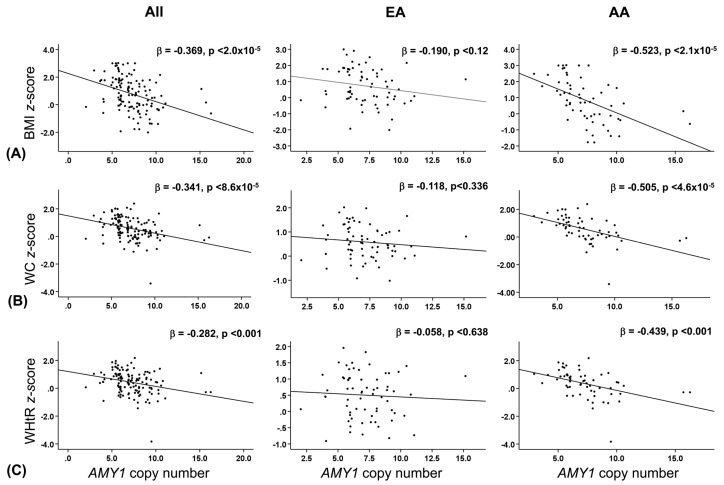
Relationship between *AMY1* copy number and obesity measurements. Association of *AMY1* copy number with (**A**) BMI *z*-score, (**B**) WC *z*-score, and (**C**) WHtR *z*-score in European American, African American, and study population. Standardized β-coefficient value was used to quantify the association. When considering interaction, a *p* value < 0.05 is significant.

We next analyzed the association between *AMY1* copy number and BMI *z*-score, waist circumference *z*-score (WC *z*-score), and waist:height ratio *z*-score (WHtR *z*-score) (Figure 3). Linear regression analysis showed a significant inverse association between *AMY1* copy number and BMI *z*-score in the whole study population (β co-efficient; −0.369, *p* < 2.0 × 10^−5^) (Figure 3A). Similarly, a significant inverse association trend was observed between *AMY1* copy number and both WC *z*-score (β co-efficient; −0.341, *p* < 8.6 × 10^−5^) and WHtR *z*-score (β co-efficient; −0.282, *p* < 0.001) (Figure 3B,C).

Race specific linear regression analysis between *AMY1* copy number and BMI *z*-score showed that AA (β co-efficient; −0.523, *p* < 2.1 × 10^−5^) had a greater significant inverse association compared to EA (β co-efficient; −0.190, *p* < 0.12) (Figure 3A). AA also had a greater significant inverse association between *AMY1* copy number and both WC *z*-score (β co-efficient; −0.505, *p* < 4.6 × 10^−5^) and WHtR *z*-score (β co-efficient; −0.439, *p* < 0.001) compared to EA (β co-efficient; −0.118, *p* < 0.336 and β co-efficient; −0.058, *p* < 0.638, respectively) (Figure 3B,C).

## 4. Discussion

This present study explored the association between the obesity measurements and *AMY1* copy number in elementary school children aged 6–10 years old with normal weight and overweight/obese. The median copy number of *AMY1* was less in obese children compared to normal weight children. We also found that the *AMY1* copy number was negatively associated with the obesity measures such as BMI *z*-score, waist circumference *z*-score, and waist circumference adjusted height *z*-score. These results correspond to another study reported with Mexican children, which suggested that normal weight participants had an *AMY1* copy number greater than 10 and a higher copy number reduced the risk of obesity [29]. Increased BMI was also found to be associated with a low *AMY1* copy number and decreased level of salivary amylase in European and Asian adults [23]. A genetic link between obesity and carbohydrate metabolism has been reported [23]. Patients with obesity, type 1 and 2 diabetes, and metabolic syndrome have been shown to have lower serum salivary amylase levels [35]. The copy number of serum and salivary *AMY1* and *AMY2* is lower in patients with metabolic syndrome [23,27,36]. Normal weight adults with high *AMY1* copy number have shown improvements in the glycemic control [37].

We also assessed the impact of race and found that the *AMY1* copy number was negatively associated with the obesity measures more significantly in African Americans compared to European Americans from the similar age range and region. However, there is no difference in the mean *AMY1* copy number between EA and AA as shown in Figure 2B. In another study including European American and African American children, a genome-wide study revealed a difference in some of the CNVs and showed the genetic vulnerability of common childhood obesity in the participants [38]. Viljakainen et al., in a study based in Finland, showed that there was no difference in *AMY1* copy number between healthy and obese participants aged 15–25 years but only the obese women had a lower copy number compared to healthy participants [27]. Therefore, a large discrepancy exists in people from different locations and within a specific population. The difference is mainly due to adaptation to different dietary habits and environmental factors. Several nutrition education programs are working to reduce and prevent obesity by promoting a healthy diet, limiting sweetened beverages, and increasing physical activity in schools in Alabama [39,40]. The limitation of this study is the small number of participants on which the results are based. The dietary habits, environmental factors, socioeconomic status, and parental influences that might influence copy number are also not included.

In summary, we found the *AMY1* copy number was significantly decreased in obese compared to normal weight children. A significant inverse association between obesity measurements and *AMY1* copy number was observed. African Americans have a stronger association between low *AMY1* copy number and obesity compared to European Americans. 

## Figures and Tables

**Table 1:** General characteristics of the study population.

	All	Normal Weight (NW)	Overweight/Obese (OW/OB)	*p* Value
Sex (N)	127	76	51	
Male	69	43	26	
Female	58	33	25	
Race (N)	127	76	51	
EA	68	41	27	
AA	59	35	24	
Age (years)	6.93 ± 1.79	6.80 ± 1.94	7.26 ± 1.52	
Height (cm)	131.95 ± 11.44	130.05 ± 11.07	134.80 ± 11.50	0.02
Weight (Kg)	71.05 ± 24.17	60.72 ±14.37	86.44 ± 27.54	0.00001
BMI (Kg/m^2^)	18.09 ± 3.43	16.03 ± 1.56	21.16 ± 3.15	0.00001
BMI z-score	0.77 ± 1.22	−0.03 ± 0.78	1.99 ± 0.59	0.00001
Waist circumference (cm)	64.61 ± 8.62	60.28 ± 5.26	71.06± 8.67	0.00001

**Table 2:** Descriptive analysis of *AMY1* copy number variations (CNV) in study population.

Groups	Mean	N	Standard Deviation	Median	Minimum	Maximum	Range
NW	7.9650	76	2.34632	7.8350	2.03	16.25	14.22
OW/OB	6.2076	51	1.48473	5.8900	2.95	10.46	7.51
Total	7.2593	127	2.21353	6.9300	2.03	16.25	14.22

**Table 3:** Race specific descriptive analysis of *AMY1* CNV.

Groups	Mean	N	Standard Deviation	Median	Minimum	Maximum	Range
White/European American (EA)	7.1684	68	2.07064	6.8950	2.03	15.16	13.13
African American (AA)	7.3641	59	2.38129	6.9400	2.95	16.25	13.30
Total	7.2593	127	2.21353	6.9300	2.03	16.25	14.22
